# Stakeholders' Experiences and Perspectives of Patient and Public Involvement (PPI) in Maternal and Neonatal Clinical Trials: A Qualitative Evidence Synthesis

**DOI:** 10.1111/hex.70495

**Published:** 2025-11-26

**Authors:** Kathleen Hannon, Jessica Eustace‐Cook, Déirdre Daly, Valerie Smith

**Affiliations:** ^1^ School of Nursing and Midwifery Trinity College Dublin, The University of Dublin Dublin Co. Dublin Ireland; ^2^ Health Research Board ‐ Trials Methodology Research Network (HRB‐TMRN) University of Galway Galway Co. Galway Ireland; ^3^ The Library of Trinity College, Trinity College Dublin The University of Dublin Dublin Co. Dublin Ireland; ^4^ Trinity Centre for Maternity Care Research (TCMCR) Trinity College Dublin, The University of Dublin Dublin Co. Dublin Ireland; ^5^ School of Nursing, Midwifery and Health Systems University College Dublin Dublin Co. Dublin Ireland

**Keywords:** maternity trials, neonatal trials, Patient and Public involvement (PPI), qualitative evidence synthesis, thematic synthesis

## Abstract

**Introduction:**

While there is a growing emphasis on Patient and Public Involvement (PPI) within maternal and neonatal research, there is a lack of evidence on how PPI is currently implemented. The aim of this qualitative evidence synthesis was to gain insight into stakeholders' experiences and perspectives of PPI in maternal and neonatal trials.

**Methods:**

CINAHL, MEDLINE, PsycINFO, EMBASE, Web of Science, and Maternity and Infant Care (OVID) were searched from inception dates to September 2023, supplemented by a search of Google Scholar. Eligibility screening and quality appraisal of each included record were independently undertaken by two reviewers. Data extracted from the included records were thematically synthesised using Thomas and Harden's approach.

**Results:**

Nine records were identified as eligible and included in the review. Three themes were generated from the synthesis, each with two or three subthemes. The main themes were ‘building a successful PPI partnership’, ‘impact of PPI on trial design and development’, and ‘impact of PPI on stakeholders’. Of 15 discrete findings, 1 was assessed as having high confidence, 11 as moderate and 3 were assessed as having low confidence.

**Conclusion:**

Researchers who facilitated PPI described it as a positive and enjoyable experience that improved trial design and considered PPI as an asset to research. However, there was a lack of information available on PPI contributors' first‐hand perspectives or experiences, which considerably limits our understanding of their opinions on how PPI is currently conducted.

**Protocol Registration:** The protocol for this qualitative evidence synthesis was registered on PROSPERO (PROSPERO registration number CRD42023383878).

## 1 Introduction

While Patient and Public Involvement (PPI) has become a research priority globally [1], the frequency of the occurrence of PPI within research often varies by country [2, 3, 4].

Involving parents and other PPI contributors, such as adults who were born preterm, in the design of neonatal studies has been considered an effective way to conduct research that is acceptable and relevant to the needs of newborns and their families [5, 6]. However, involving PPI contributors in designing and conducting neonatal research is considered to be somewhat of a recent development [7, 8, 9]. In contrast, an early study on PPI suggested that PPI was prominent in maternity health research due to the numerous organisations advocating for pregnant and postpartum women's health needs [10]. Despite this report, there appears to be limited information on how PPI is being implemented within maternal health research.

Some researchers have expressed that there may be unique challenges when recruiting PPI contributors for maternal health research studies as it may involve recruiting women who are either pregnant or early postpartum when, during this time period, maternal research may feel at it most relevant to potential contributors [11, 12]. As a result, this may place limits on the time and opportunities that researchers have to identify and recruit PPI contributors [11, 12].

As clinical trials are an integral component of establishing evidence‐based maternal and neonatal care [13, 14, 15], this review sought to explore the views and experiences of those involved in PPI within maternal and neonatal trials. The objective of this qualitative evidence synthesis (QES) was to synthesise the available literature on the experiences of stakeholders involved in PPI at any stage of a maternal trial or neonatal trial.

## 2 Materials and Methods

The QES protocol was registered on PROSPERO (CRD42023383878) and is published [16, 17]. The QES adheres to the Enhancing Transparency in Reporting the Synthesis of Qualitative Research (ENTREQ) statement [18] (Appendix S1).

### 2.1 Inclusion Criteria

We included all primary qualitative research of any design and mixed‐methods research where qualitative data could be extracted, that detailed stakeholders' perceptions and experiences of PPI in maternal and neonatal clinical trials. Stakeholders included ‘trial participants, PPI contributors, and any member of the trial research team (e.g., principal investigators, research midwives/nurses, trial managers, and statisticians)’ [16].

### 2.2 Search Strategy

As varied terminology is used to refer to PPI, with terms such as participation, engagement, and involvement used interchangeably [19, 20], a broad search strategy was necessary (Appendix S2). CINAHL, MEDLINE, PsycINFO, EMBASE, Web of Science, and Maternity and Infant Care (OVID) were searched from inception dates to October 2023, supplemented by a search of Google Scholar for grey literature. No language restrictions were applied.

A prior decision was taken for at least two reviewers to independently screen 20% of the records (*n* = 4113) by title and abstract, with the understanding that if a minimum of 80% agreement was not achieved on these records, as per AMSTAR 2's recommendation [21], then *all* records would require independent screening at title and abstract. As agreement of 97.3% was achieved, one reviewer (K. H.) proceeded to screen the remaining records, forwarding any potentially eligible record for full text review. Screening of full text records was conducted independently by a minimum of two reviewers.

### 2.3 Quality Assessment

The Evidence for Policy and Practice (EPPI)‐Centre's appraisal tool was used to assess the quality of the included records [22]. The tool consists of 12 items across three categories which assess the quality of reporting of study methods, strategies for establishing reliability and validity, and the extent to which findings are rooted in the participant's experience. Each included study was assessed as to whether each item was met, partially met or not met. Quality assessment was conducted independently by two reviewers, who then compared their assessments and reached consensus through discussion. All eligible records were included in the QES, irrespective of methodological quality.

### 2.4 Data Extraction and Synthesis

A purposively designed form was used to extract data. Extracted data included information on the aim of the record, trial details, study methods and any information on PPI, including participant quotations and authors' interpretations (Appendix S3). Each record was also uploaded to NVivo 14 [23], where thematic synthesis was conducted inductively following Thomas and Harden's [24] approach. This consisted of: (1) coding extracted data line by line, (2) the generation of descriptive themes and (3) the generation of analytical themes from the descriptive themes [24]. Once all records had been read and coded line by line, the codes generated were checked for similarities to group them into descriptive themes.

Appendix S4 presents the audit trail of themes developed from codes to descriptive themes and then analytical themes. As thematic synthesis is also typically used to explore individuals' beliefs and experiences in qualitative syntheses [25], it was considered an appropriate method to achieve the objectives of this particular QES.

All stages of thematic synthesis were undertaken by one reviewer (K. H.) and corroborated independently by two reviewers (D. D. and V. S.) who reviewed the extracted and coded data. Frequent communication between the reviewers resulted in the refinement of the generated themes. Reflexivity is an important element when conducting qualitative research and involves considering how the researcher's views and perspectives can influence how research is conducted and analysed [26]. The reviewers are maternal health researchers with backgrounds in sociology (K. H.), midwifery (D. D. and V. S.), and in conducting both maternal and neonatal trials (D. D. and V. S.). All acknowledge that they are advocates of women's health, PPI in research, and participatory approaches in research design and conduct.

### 2.5 Assessment of Confidence in the Findings

The GRADE‐CERQual (Confidence in the Evidence from Reviews of Qualitative Research) approach was used to assess confidence in the QES findings [27, 28, 29, 30, 31]. Assessing confidence consists of formulating the findings into short statements that provide a clear description of each review finding and then assessing the confidence in each statement based on four components: methodological limitations, data coherence, data adequacy, and relevance of the included records to the overall review question [32]. Following assessment of each component, an overall assessment is established, ranging from high, moderate, low, to very low confidence [31].

Following the GRADE‐CERQual working group's guidelines, each finding was assumed to be of ‘high confidence’, and was subject to downgrading according to the individual components' assessment [31]. K.H. conducted the assessments, which were reviewed by at least one other reviewer to enhance rigour and reduce subjectivity in this process. Consensus was achieved through discussion.

## 3 Results

**Figure 1:**
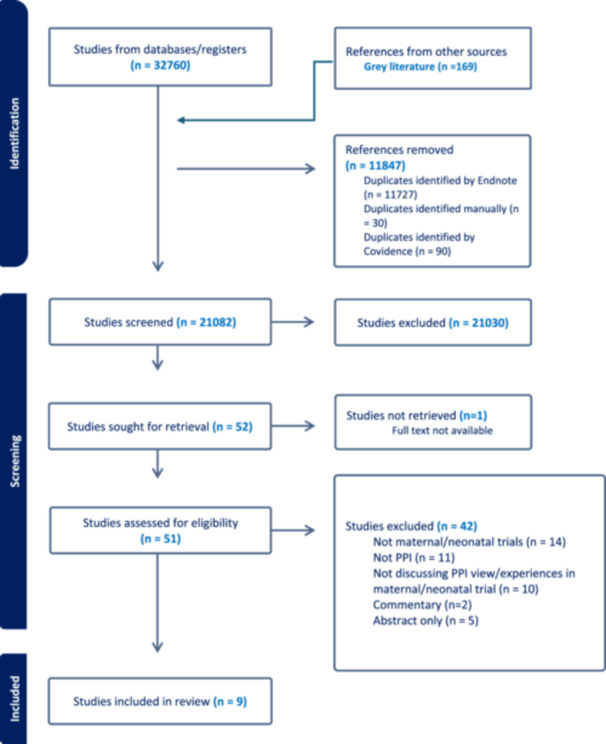
PRISMA flowchart [33], produced using COVIDENCE [34].

Figure 1 illustrates the search and selection process. From an initial retrieval of 32,929 records, and screening following removal of duplicates (*n* = 11,847), 21,082 records were screened, and nine records published between 2015 and 2023 were included in the QES (Figure 1). Two of the records reported on the same randomised controlled trial (RCT), ‘COLLABORATE’ [35, 36], with one record reporting on COLLABORATE in combination with two other interventions [36]. Table 1 presents the summary characteristics of the included records.

**Table 1:** Characteristics of included records.

Reference	Location and setting	Type of record	Trial category and design	Condition of interest	Aim of the study	Information provided on PPI activity
Lammons et al. [35]	UK, virtual	Qualitative	Neonatal Double‐cluster RCT	Preterm nutrition	To report on PPI conducted to design an RCT.	− Focus groups/Interviews: 20 volunteers (10 clinicians, 7 parents, 2 former patients, and 1 parent/former patient) − Focus groups: Parent‐patient groups (*n* = 9), all women (7 parents, 1 former patient, and 1 parent who is also a former patient).
Moss et al. [36]	UK, virtual	Qualitative	Neonatal two RCTs and Cross‐sectional survey/questionnaire	Preterm nutrition	To report PPI consultations across three studies (two RCTS, one questionnaire)	− 23 contributors (parents and adults born preterm)
Levene et al. [37]	UK, virtual	Report	Neonatal RCT	Breastmilk feeding, preterm birth	To report on PPI conducted to design an RCT.	− PPI survey – 675 parents. − Online PPI panel – Six mothers − Interactive exercise − 12 contributors.
Morgan et al. [38]	UK, Two mother and baby groups	Qualitative	Maternal	Smoking during pregnancy and breastfeeding	To report on PPI and qualitative research conducted to develop a trial intervention.	− Mothers, grandmothers, and families
						Two formal groups:
						− Group 1: 9 contributors − Group 2: 12 contributors
Onukwugha et al. [39]	UK, virtual	Qualitative	Maternal Future pilot RCT	Alcohol consumption in pregnancy	To assess maternity service users' and midwives' experiences of co‐creating an intervention.	Five online workshops evaluating experiences of co‐creating an intervention:
						− Maternity service users (*n* = 6) − Midwives (*n* = 13)
Patel [40]	UK	Opinion piece with embedded case study	Neonatal	Prematurity (withholding milk feeds – blood transfusion)	Case study – Opinion on PPI in neonatal trials	Case study, with opinions on PPI provided by:
						− PPI contributor − Clinical trial investigator
Rayment et al. [41]	UK, Mother and baby groups	Qualitative	Neonatal	Preterm birth	To describe how women contributed to designing a neonatal trial.	− 35 mothers of young children
Silver et al. [42]	US, Phone interviews	Qualitative	Maternal‐ Neonatal Quasi‐experimental	Prevention of infant obesity	To describe stakeholder engagement in RCT development	− One father advocate and five representatives from five relevant organisations on a Community Advisory Board (12 members)
Timm et al. [43]	Denmark, home interviews	Qualitative	Maternal	Gestational diabetes mellitus (GDM)	To describe how co‐creation developed an intervention for an RCT	− Women with prior GDM and their families

### 3.1 Definitions of Used Terms

Seven included records were articles written by researchers describing how they incorporated PPI in the development of a trial [35, 36, 37, 38, 41, 42, 43]. For coherency, we refer to these authors as ‘researchers’ throughout this review. The two remaining included records included one case study where a PPI contributor and a clinical investigator provided their views on PPI in neonatal trials [40] and the results of an evaluation of PPI contributors' and midwives' experience of co‐creating an intervention together [39]. There were several terms used within the included records to describe lay individuals who participated in PPI, such as ‘research partners’ [38] or ‘parent–patient participants’ [35]. For clarity, we refer to them as ‘PPI contributors’ in this review. Just two of the included records reported first‐hand accounts from PPI contributors on their views on PPI [39, 40]. In an additional two records, researchers reported *on* PPI contributors' experience of PPI [36, 38], for instance, reporting that PPI contributors expressed ‘pride’ regarding their impact on the study [38] (p. 22).

### 3.2 PPI Activity Reported in the Included Records

All of the included records reported conducting PPI in the early stages of planning and designing a trial, including providing input into the trial's aims and proposed design [35, 36, 38, 39, 41, 42], developing and reviewing trial documents such as Participant Information Leaflets (PILs) [35, 36, 37, 40], and developing elements of the proposed intervention [39]. Included records also gathered information on contributors' experiences of the condition of interest, such as gestational diabetes mellitus (GDM) and breastfeeding, to inform the trial design and identify key outcomes [37, 43]. None of the records provided information on training opportunities or reimbursement for PPI contributors' time.

### 3.3 Quality Assessment Results

None of the included records met all 12 of the items in the quality assessment tool. Five studies met eight items [36, 37, 38, 39, 42], one of which partially met a further four items [38], and another partially met another three items [42]. The results of the quality assessment for all included studies are presented in Appendix S5.

### 3.4 Thematic Synthesis

Three analytical themes were generated, within which there were two, three and two sub‐themes, respectively. The analytical themes were ‘building a successful PPI partnership’, ‘impact of PPI on trial design and development’, and ‘impact of PPI on stakeholders’.

### 3.5 Theme 1: Building a Successful PPI Partnership

Six records contributed data relating to the process of ‘building a successful PPI partnership’. Actions by researchers taken to facilitate PPI and to build a relationship with PPI contributors were central to this theme, which included two subthemes: ‘facilitating engagement in PPI’, and ‘relationships of PPI’.

#### 3.5.1 Facilitating Engagement in PPI

Researchers employed strategies to initiate and sustain PPI contributors' involvement, particularly when working with members of underrepresented populations who were unfamiliar with PPI [37, 38, 41, 43], by meeting contributors on their own ‘turf and terms’ [38] (p. 6).

Attempts were made to accommodate PPI contributors as parents with competing responsibilities of caring for young children, such as scheduling evening meetings [37, 43] and incorporating PPI into contributors' routine schedules [38, 41]. Other strategies included using online meetings, online surveys, and email communication [37, 39]. However, online PPI sometimes resulted in technological difficulties or inhibited rapport [39].

Researchers chose to work with pre‐existing mother and baby groups as their PPI contributors [38, 41]. This facilitated researchers to reach PPI contributors from underrepresented groups, whose participation was sought out as a demographic disproportionately impacted by the trial's condition of interest, such as preterm birth [38, 41]. Travelling to meet contributors fostered trust between researchers and PPI contributors [38].

We were able to involve a diverse sample of women by making contact with existing community‐based groups… rather than requiring women to attend a focus group in an unfamiliar venue.

(Rayment et al. [41], p. 8)

#### 3.5.2 Relationships of PPI

Five records explored the relationships forged among PPI contributors and the relationships developed between researchers and PPI contributors [36, 37, 38, 39, 41]. Although some researchers described the relationship between PPI contributors as friendly and mutually collaborative [38, 41], others reported that not all contributors participated equally, or that dominant voices or conflict emerged [38, 39]. While researchers recognised that alternative or disconfirming views should be sought [19, 38], it was sometimes difficult to reach an agreement [37, 38, 39, 43].

There was very little consensus …. and no single type of response was given by more than 10% of respondents.

(Levene et al. [37], p. 5)

Researchers also reflected on power dynamics, with most researchers describing how their PPI approach challenged power relations [36, 38], or that all stakeholders' views were equally valued [39, 41]. However, one acknowledged that researchers maintained control over how PPI was conducted and, subsequently, its capacity to influence trial design [37].

There was an unequal balance of power with PPI contributors unable to ensure that their concerns were acted upon in any areas of tension or disagreement.

(Levene et al. [37], p. 11)

While two researchers led the PPI activity in one study [38], there was a lack of interest from both PPI contributors and the wider research team to engage with each other.

… when research staff team members were invited to attend visits to the mother and baby groups [PPI group], the idea was mostly rejected. Some made highly sceptical comments like ‘you'll be lucky!’ and ‘ahem, no! [laughing]’

(Morgan et al. [38], p. 13)

### 3.6 Theme 2: Impact of PPI on Trial Design and Development

All records contributed data on the impact of PPI on trial design and outcomes, most notably on the language used in trial documents. This theme had three subthemes: ‘addressing emotional needs’, ‘minimising burden of trial participation’, and ‘PPI contributors are a unique asset to research’.

#### 3.6.1 Addressing Emotional Needs

PPI contributors' views were regularly situated in their own experience of stressful and potentially traumatic events, such as being the parents of a preterm baby who stayed in a neonatal intensive care unit (NICU) or having complications during pregnancy and birth [35, 37]. PPI contributors discussed that being a parent, particularly as a mother, of a baby in a NICU or experiencing difficulties breastfeeding could evoke feelings of inadequacy and guilt [35, 36, 37]. PPI contributors described the environment that potential trial participants and parents found themselves in as stressful and anxiety‐inducing and advocated for sensitivity towards the emotional state of participants/guardians when designing a trial [35, 36, 37, 40].

A key component of ensuring this was to improve the clarity and sensitivity of the language used in trial documents. PPI contributors made changes to trial language to avoid parents who are being invited to take part in a trial and participants experiencing unnecessary anxiety or confusion [35, 36, 38, 40, 41].

The language used can also help avoid making mothers feel inadequate by recognising the challenges of providing milk for their babies and alleviating the pressure to breastfeed.

(Lammons et al. [35], p. 5)

PPI contributors championed the use of empathetic, respectful language that recognised the individual difficulties that mothers and fathers may be experiencing at the time of trial recruitment [35, 36, 37]. Contributors emphasised ways in which the trial design could help make fathers feel integral to the family unit [36, 42].

[PPI contributors] felt that mothers' tasks such as expressing milk or nursing were ‘closer’ to the baby, while absorbing data allowed fathers to combat feelings of helplessness and fulfil their ideas of stereotypical masculine ‘protection’.

(Moss et al. [36], p. 2)

#### 3.6.2 Minimising Burden of Trial Participation

PPI contributors queried whether a proposed trial placed unnecessary strain on parents of trial participants and focused on reducing the burden of trial participation [35, 36, 37]. For example, PPI led to the provision of both short and long versions of the PIL so that parents could choose the amount of trial information they engaged with [35, 36, 37]. Researchers perceived the changes made to the trial as a result of PPI input as having the ability to improve trial recruitment [36, 40, 41].

Some respondents reported a concern that the research study would be an extra burden to participants, taking time away from their baby and intruding into their lives at a stressful time.

(Levene et al. [37], p. 6)

PPI contributors encouraged researchers to promote the trial as a partnership between clinicians and parents, with the intent that women who participated in the trial, or the parents of neonates, would feel valued and actively involved [35, 36, 37, 42].

Our community stakeholders encouraged an inclusive culture that engages fathers from the start.

(Silver et al. [42], p. 13)

#### 3.6.3 PPI Contributors Are a Unique Asset to Research

Researchers described PPI contributors as providing a unique asset to research that could not be replicated through conducting qualitative research [38, 42]. The informality and environment in which PPI was conducted was viewed as conducive to open discussion that might not be as easily replicated through qualitative interviews or focus groups [38].

While researchers recognised PPI's value [20, 38, 41, 42], there was also the perception of a distinct difference between PPI contributors' experiential knowledge and researchers' professional knowledge [35, 38, 40, 41]. Contributors' views were considered to be reflective of the wider target population, which provided researchers with the ability to identify issues which could impede trial recruitment that had not previously considered [41]. For example, contributors highlighted the potential for women to have reservations about participating in a trial involving probiotic consumption during pregnancy, as women may perceive the probiotics as medication due to their being administered in tablet form [41]. Alternatively, as contributors were involved in trial development based on their own experiential knowledge, this was viewed as a limitation [38]. As PPI contributors did not have the same research training nor the professional experience as established researchers, it was suggested that this restricted the roles that PPI contributors can assume in trial development and conduct [38].

PPI contributors… may not consider reflexivity and bias in the same way/s as trained researchers, which may limit the role they have and stages in which it is appropriate for them to be involved.

(Morgan et al. [38], p. 19)

Researchers considered PPI contributors' views to be both reflective of their target population and somewhat indicative of a vast difference between the views of researchers and lay individuals. Researchers positioned their own views as grounded in a ‘rationalistic’ worldview [41] (p. 8), distinct from more complicated views that nonresearchers might hold.

The ability of humans to maintain and act on such complex, apparently contradictory views may be underestimated by researchers, who often draw on a more linear and rationalistic model of human decision‐making.

(Rayment et al. [41], p. 8)

### 3.7 Theme 3: Impact of PPI on Stakeholders

The theme of ‘impact of PPI on stakeholders’ comprised researchers' personal reflections on how they experienced PPI or reporting back on other stakeholders' experiences [38, 39, 40, 41, 43]. This theme comprised two subthemes: ‘PPI as an enjoyable experience’ and ‘impact on PPI contributors’. Although the theme consisted of relatively thin data, it provides important insight into how contributors felt about PPI and the personal gains that could be attained.

#### 3.7.1 PPI as an Enjoyable Experience

PPI was discussed in positive terms and described as an enjoyable experience [36, 39]. Consultations were described as not an ‘onerous’ endeavour [37] (p. 3), and co‐creation was described as ‘productive and rewarding’ [39] (p. 1). This was more apparent when sensitive topics were covered. In a trial investigating alcohol use in pregnancy, a PPI contributor described PPI as a cathartic experience, allowing her to make ‘peace with myself’ [39] (p. 3).

The midwives and maternity service users enjoyed taking part in the workshops and good humour, openness, and honesty dominated the discussions.

(Onukwugha et al. [39], p. 3)

#### 3.7.2 Impact of PPI on Contributors

Researchers perceived that PPI contributors felt pride and accomplishment from taking part [38, 39] (p. 3), with contributors expressing approval that their involvement was acknowledged in the dissemination of findings [38]. The willingness of women to continue participating in future PPI and advocacy work was taken by researchers as evidence that PPI contributors found the experience to be valuable [38, 39, 41]. However, not all contributors showed interest in continuing their involvement in clinical research [43].

This accessible approach also engaged the women's interest and enthusiasm for research, reflected in the number of women (*n* = 9) from a range of ethnic backgrounds who volunteered to be contacted and maintain an ongoing involvement as user representatives.

(Rayment et al. [41], p. 8)

### 3.8 GRADE‐CERQual Assessment

The CERQual Evidence profile is presented in Appendix S6. Of 15 discrete findings' statements that were formulated in the synthesis, only one was assessed as having high confidence. This statement related to the impact PPI had on improving the clarity and sensitivity of the language used within the trial, notably in PILs, to avoid causing anxiety or confusion for trial participants and parents. Of the remaining 14 findings' statements, 11 were assessed as having moderate confidence and 3 findings as having low confidence.

## 4 Discussion

With the ongoing integration of PPI into research across Europe and beyond [3], it has been acknowledged that there are challenges in recruiting PPI contributors, as well as uncertainty in how contributors can be involved in research [7, 44]. As PPI contributors, women and their families offer researchers a strong resource of experiential knowledge that could, potentially, benefit the development and conduct of maternal and neonatal trials. For example, recruitment and retention of trial participants are ongoing challenges for trialists [45, 46]. While a significant factor influencing the decision to participate in both maternal and neonatal trials is the perception of an intervention's risks or benefits posed to the child [14, 47, 48, 49], another factor positively influencing pregnant women's decision‐making was that trial participation was perceived as an easy and straightforward task [47]. Convoluted medical terminology was reported as a factor that would dissuade women from participating in clinical research during pregnancy [47].

Our review provided examples of where PPI contributors, drawing on their own lived experiences, can make changes to a trial design that could positively influence trial recruitment and retention rates, for example, by (1) avoiding potential negative responses when potential participants or parents of neonates first encounter trial information or (2) from discouraging trial participants' continued involvement through unnecessary trial burden.

Researchers who conducted PPI in a neonatal research project discussed the difficulties of choosing a role or activity that contributors would find both achievable to complete *and* have meaningful involvement [9]. In eight of the records included in this QES, PPI occurred during the planning stages of a maternal or neonatal trial. Reportedly, PPI in trials typically involves contributors reviewing or developing PILs, as seen in the QES [50, 51], or participating in a more formal capacity, for example, via trial steering committee membership [52]. While strategies of engaging and involving PPI contributors continue to evolve, it may be helpful to explore how contributors wish to be involved.

The QES found that there was some variation in researchers' attitudes to the insights that PPI contributors can provide. Albeit a finding with limited data to support it, researchers perceived PPI contributors' views as distinct from the research‐oriented perspectives of researchers and suggested that this was a potential limitation on the role of PPI contributors in trial research due to their lack of professional research experience. This belief in contributors' limited capability to take part in research could be a bias with unintended consequences on how PPI contributors are involved in trials.

The included records did not expand on some of the limitations that the researchers encountered when including PPI in trials. For instance, when PPI contributors did not respond to invites to participate in formal team meetings or to take part in future PPI activity, it was not clear whether the researchers had sought an explanation. This could result in key insights on potential barriers to PPI remaining unidentified.

This study has both strengths and limitations. One key strength is the volume of records (*n* = 21,082) that were screened for inclusion in this QES. Only nine records met the inclusion criteria, which points to an important gap in reporting PPI activities in trials and PPI contributors' experiences. While we implemented a broad search strategy to capture all potentially eligible records, it is possible, due to the varied terminology used to describe PPI in trials, that some potentially eligible records may not have been captured. However, due to the limited number of eligible records identified from a substantial number of retrieved records, we believe this is unlikely. The limited records eligible for inclusion meant that some of the themes generated lack ‘rich’ data and limited the ability to move beyond descriptive to analytical themes. As seen in the GRADE‐CERQual assessment, many of the themes were found to be of moderate or low confidence, often due to methodological limitations of the included records and/or issues relating to the adequacy of the data.

The identified scarcity of PPI contributors' direct first‐hand accounts of their experiences and perspectives also shows a critical gap in knowledge, especially in understanding what works well and what processes could be improved. The majority of the included records were aimed at detailing how PPI was conducted and its impact on study development, as opposed to reporting on PPI contributors' experiences of involvement.

While researchers do not require ethical approval to conduct PPI, ethical approval would likely be required if researchers wished to collect and report data on stakeholders' views and experiences of PPI in a particular trial, and it is possible that this had an impact on the available information in the published records included in this QES. A consequential limitation of the minimal direct first‐hand data from PPI contributors is that it is difficult to assess whether PPI contributors' experiences of the PPI process are reflective of those reported by the researchers.

As the findings highlighted that PPI contributors provide unique insights into trial design and development, it would therefore be reasonable to extrapolate that contributors' insights into the process of PPI itself would also be illuminating for the wider research community.

As seen in the GRADE‐CERQual assessment, many of the themes were found to be of moderate or low confidence, often due to methodological limitations of the included records and/or issues relating to the adequacy of the data.

## 5 Conclusion

Researchers reflected on the outcome of their approach to building a successful PPI partnership, the impact that PPI had on improving trial documentation and design for the benefit of trial participants, and shared their perception that PPI was a positive experience for both researchers and PPI contributors. However, with minimal direct data from PPI contributors, it is difficult to gauge whether the views of researchers, particularly the belief that PPI contributors were valued members of the research team, are a commonly shared experience by PPI contributors.

## Author Contributions

**Kathleen Hannon:** conceptualisation, methodology, investigation, formal analysis, validation, data curation, visualization, writing – original draft, writing – review and editing. **Jessica Eustace‐ Cook:** methodology. **Déirdre Daly:** conceptualisation, methodology, investigation, formal analysis, validation, writing – review and editing. **Valerie Smith:** conceptualisation, methodology, investigation, formal analysis, validation, funding acquisition, writing – review and editing.

## Ethics Statement

Ethics approval was not required for this review of previously published material.

## Consent

The authors have nothing to report.

## Conflicts of Interest

The authors declare no conflicts of interest.

## Patient and Public Involvement

Patients and members of the public were not involved in conducting this review. This review was conducted to inform a qualitative study with PPI contributors and trial team members in maternal and neonatal trials. The qualitative study involved the input of members of the public in its development.

## Supporting information


**Appendix 1:** ENTREQ reporting guideline [ 18].

**Appendix 2:** Search strategy.

**Appendix 3:** Data extraction form.

**Appendix 4:** Development of themes and sub‐themes, following Thomas and Harden's (2008) thematic synthesis approach [ 24].

**Appendix 5:** Quality assessment using EPPI‐Centre's appraisal tool for assessing quality criteria [ 22].

**Appendix 6:** GRADE‐CERQual assessment [ 27, 28, 29, 30, 31, 32].

## Data Availability

Data are available from the corresponding author upon reasonable request.
