# Supplementary material for: Stakeholders' Experiences and Perspectives of Patient and Public Involvement (PPI) in Maternal and Neonatal Clinical Trials: A Qualitative Evidence Synthesis
Source: Health Expect. 2025 Nov 26;28(6):e70495. doi: 10.1111/hex.70495 (PMC12657262; doi:10.1111/hex.70495)
Supplement: Supplementary file 2 — Appendix 2: Search strategy. [file HEX-28-e70495-s002.docx]

# Appendix 2: Search strategy

| **Concept** | **Index Terms and keywords** |
| --- | --- |
| Concept 1: Patient and public involvement | **Index Terms:**  EMBASE: ‘patient participation’/exp OR ‘stakeholder engagement’/exp OR ‘patient and public involvement’/exp  CINAHL: (MH "Consumer Participation")  MEDLINE (EBSCO): (MH "Patient Participation") OR (MH "Community Participation+")  PsycInfo: DE "Community Involvement" OR DE "Client Participation"  **Keywords:**  (public* OR citizen* OR patient* OR user* OR "service user*" OR client* OR "care giver*" OR caregiver* OR carer* OR "lay member*" OR stakeholder* OR consumer* OR representative* OR community) N2 (engag* OR involv* OR participa* OR consult* OR activat* OR partner* OR collab* OR contribut* OR "co-develop*" OR "co-research*" OR "co-lead*" OR "co-produc*" OR "decision-making" OR advisor*" OR "shared leadership")  OR  TI/AB (ppi* OR "patient involvement*" OR "public involvement*" OR "patient engagement*" OR "public engagement*" OR "patient particip*" OR "citizen science*") |
| Concept 2: Clinical trials in maternal and neonatal health | **Index Terms:**  EMBASE: ('clinical trial'/exp OR 'clinical trial (topic)'/exp) AND ('maternal care'/exp OR ‘obstetrics’/exp OR 'pregnancy'/exp OR 'pregnant woman'/exp OR 'newborn'/exp OR 'childbirth'/exp OR 'perinatal care'/exp OR 'prenatal care'/exp OR 'puerperium'/exp OR 'infant'/exp)  CINAHL: ((MH "Clinical Trials+") AND ((MH "Obstetrics") OR (MH "Pregnancy+") OR (MH "Expectant Mothers") OR (MH "Infant, Newborn+") OR (MH "Childbirth+") OR (MH "Perinatal Care") OR (MH "Prenatal Care") OR (MH "Puerperium") OR (MH "Infant+"))  MEDLINE: (MH "Clinical Trials as Topic+") AND ((MH "Maternal Health") OR (MH "Obstetrics") OR (MH "Pregnancy+") OR (MH "Pregnant Women") OR (MH "Infant, Newborn+") OR (MH "Parturition+") OR (MH "Perinatal Care+") OR (MH "Prenatal Care") OR (MH "Postpartum Period+") OR (MH "Infant+"))  PsycInfo: DE "Clinical Trials" AND ((((((((DE "Obstetrics") OR (DE "Pregnancy")) OR (DE "Prenatal Care")) OR (DE "Intrapartum Period")) OR (DE "Antepartum Period")) OR (DE "Postnatal Period")) OR (DE "Birth")) OR (DE "Neonatal Period"))  **Keywords:**  ("clinical trial* OR trial* OR intervention*) AND (matern* OR obstetric* OR labour OR labor OR pregnant* OR "pregnan* wom*n" OR "expect* mother*" OR birth* OR childbirth* OR "child birth" OR antenatal* OR "ante-natal*" OR antepartum OR ante-partum OR intrapartum OR prenatal* OR "pre-natal*" OR perinatal* OR postpartum* OR puerperium OR parturition OR "post-partum*" OR neonatal* OR neonat* OR newborn* OR "new-born*" OR infant*) |
