# Supplementary material for: Stakeholders' Experiences and Perspectives of Patient and Public Involvement (PPI) in Maternal and Neonatal Clinical Trials: A Qualitative Evidence Synthesis
Source: Health Expect. 2025 Nov 26;28(6):e70495. doi: 10.1111/hex.70495 (PMC12657262; doi:10.1111/hex.70495)
Supplement: Supplementary file 3 — Appendix 3: Data extraction form. [file HEX-28-e70495-s005.docx]

# Appendix 3: Data extraction form

| **Data extraction form** | |
| --- | --- |
| Data extraction details |  |
| Extracted by | [name] |
| Reviewed by: | [name] |
| **Details regarding the paper** | |
| Author and Year |  |
| Publication Type |  |
| Conflicts of interest |  |
| Type of study |  |
| Aim of paper |  |
| **Trial details** | |
| Maternal trial/Neonatal trial |  |
| Trial details |  |
| Illness/Condition of interest |  |
| Intervention |  |
| **Details of qualitative study** | |
| Study design /  Data analysis method |  |
| Location and setting |  |
| Year study was conducted |  |
| Participant demographics |  |
| **PPI information, including data on experiences and views of PPI** | |
| Aim of PPI |  |
| Specific impact of PPI on trial design/outcomes |  |
| **Section of paper**  **[Synopsis/category]** | **Data extracted** |
| Abstract |  |
| Introduction |  |
| Methods |  |
| Results |  |
| Discussion |  |
| Appendix |  |
| **Thoughts and personal reflection** |  |
| Overall thoughts on the paper and how it contributes to the review question |  |
