# Supplementary material for: Stakeholders' Experiences and Perspectives of Patient and Public Involvement (PPI) in Maternal and Neonatal Clinical Trials: A Qualitative Evidence Synthesis
Source: Health Expect. 2025 Nov 26;28(6):e70495. doi: 10.1111/hex.70495 (PMC12657262; doi:10.1111/hex.70495)
Supplement: Supplementary file 4 — Appendix 4: Development of themes and sub‐themes, following Thomas and Harden's (2008) thematic synthesis approach [ 24]. [file HEX-28-e70495-s003.docx]

# **APPENDIX 4: Development of themes and sub-themes, following Thomas and Harden’s (2008) thematic synthesis approach [24]**

| **Example codes** | **Descriptive themes** | **Analytical sub-themes** | **Analytical themes** |
| --- | --- | --- | --- |
| Using online methods of PPI to reach parents; staff supervising contributors’ children; providing materials to prepare for PPI | Involving parents in PPI | Facilitating engagement in PPI | Building a successful PPI partnership |
| Travelling to meet PPI contributors; intentionally working with contributors not typically involved in research; | Reaching/ Involving under-represented voices |  |  |
| Reducing demands of PPI on contributors’ time; adapting PPI approach to suit contributors’ preferences; treating each PPI group as unique | Taking a responsive and flexible approach to PPI |  |  |
| Informal communication and interaction with contributors; inputting time into PPI relationship; online PPI can cause challenges to forming relationships with contributors. | Building rapport |  |  |
| PPI challenged power relations; power sharing; viewing PPI contributors as equal members of research team; PPI success perceived as dependent on the researcher; researcher in control; PPI limited to select members of wider research team | Perceptions of power | Relationships of PPI |  |
| Difficulty achieving agreement; variety of answers from contributors; researchers encouraging open expression; group dynamics and tensions (dominant voices; group ‘norms’); working with contributors new to PPI | Balancing consensus and dissent |  |  |
| Changes for participants/ parents: allow to absorb trial information at their own pace; opt-out consent; reducing intervention demands; providing option for how to be involved in trial; ensuring trial involvement suits family needs. | Reducing additional stress and anxiety | Minimising burden of trial participation | Impact of PPI on trial design and development |
| Avoiding unfamiliar terms; use of clear / neutral language; addressing language that could cause confusion or anxiety; advocating for effective communication; language influencing participation of trial parents | Clarity of language | Addressing emotional needs. |  |
| Encouraging empathetic language; removing insensitive language; avoiding inducing feelings of pressure or guilt | Sensitivity of language |  |  |
| Tone of collaboration; making fathers feel important in family unit; customization of trial involvement | Making trial participants/parents feel valued and seen |  |  |
| Emphasising need for empathy; providing emotional support services for parents; recognising emotional journey of being a parent/mother | Incorporating focus on parents’ emotional needs into trial design |  |  |
| Limitations of PPI contributors thinking process; contributors think differently than clinicians; importance of lay voice | Difference in thinking | PPI contributors are a unique asset to research |  |
| Unique insights; knowledge not found in qualitative research; unexpected feedback | Novel insights |  |  |
| PPI contributors supported trial aims and design; contributors’ views are considered reflective of target population; contributors identifying future research questions | PPI driving future research/relevant research |  |  |
| PPI described as a positive experience; PPI as an opportunity; PPI was accessible/ easy; PPI was productive; perceived value in PPI | PPI was a productive and rewarding endeavour | PPI as an enjoyable experience | Impact of PPI on stakeholders |
| Contributors engaged in PPI with intent; contributors welcomed researchers; perceived contributors as experiencing pride from involvement | Sense of pride in participation | Impact on PPI contributors |  |
| Experience ignited interest and involvement in PPI / advocacy/ research; PPI resulted in behavioural changes; resistant to change; unsuccessful attempt to get contributors to engage | Willingness to continue PPI work |  |  |
