# Supplementary material for: Stakeholders' Experiences and Perspectives of Patient and Public Involvement (PPI) in Maternal and Neonatal Clinical Trials: A Qualitative Evidence Synthesis
Source: Health Expect. 2025 Nov 26;28(6):e70495. doi: 10.1111/hex.70495 (PMC12657262; doi:10.1111/hex.70495)
Supplement: Supplementary file 5 — Appendix 5: Quality assessment using EPPI‐Centre's appraisal tool for assessing quality criteria [ 22]. [file HEX-28-e70495-s004.docx]

# **Appendix 5: Quality assessment using EPPI-Centre’s appraisal tool for assessing quality criteria [22]**

| **Reference** | **Criteria met** | **Total number of criteria fully met** | **Total number of criteria partially met** | **Description of criteria** |
| --- | --- | --- | --- | --- |
| Lammons *et al*. [35] | A,B,C,D,E,F^*^,G^*^,J,K | 7/12 | 2/12 | **Quality of reporting of study methods**  A Aims and objectives were clearly reported  B Adequate description of context of research  C Adequate description of the sample and sampling methods  D Adequate description of data collection methods  E Adequate description of data analysis methods  **Strategies for establishing reliability and validity**  ***‘Some attempt’ or a ‘good attempt’ made to establish the…***  F Reliability of data collection tools  G Validity of data collection tools  H Reliability of data analysis  I Validity of data analysis  **Extent to which findings are rooted in the participant’s experience**  J Used appropriate data collection methods for helping [participants] to express their views  K Used appropriate methods for ensuring the data analysis was grounded in the views of [participants]  L Actively involved [participants] in the design and conduct of the study |
| Moss *et al.* [36] | A,B,C,D,E,F^*^, G^*^, J, K, L | 8/12 | 2/12 |  |
| Levene *et al.* [37] | A,B,C,D,E,J,K,L | 8/12 | 0/12 |  |
| Morgan *et al*. [38] | A,B,C,D,E,F^*^,G^*^,H^*^,I^*^, J,K,L | 8/12 | 4/12 |  |
| Onukwugha *et al*. [39] | A,B,C,D,E,F^*^,G^*^,J,K,L | 8/12 | 2/12 |  |
| Patel [40] | - | 0/12 | 0/12 |  |
| Rayment *et al*. [41] | A,B,C,D,J,K,L | 7/12 | 0/12 |  |
| Silver *et al.* [42] | A,B,C,D,E,F^*^,G^*^,H^*^,J,K,L | 8/12 | 3/12 |  |
| Timm *et al.* [43] | A,B,J,K,L | 5/12 | 0/12 |  |

^*^Partially fulfilled criteria – ‘some attempt’
