# Supplementary material for: Stakeholders' Experiences and Perspectives of Patient and Public Involvement (PPI) in Maternal and Neonatal Clinical Trials: A Qualitative Evidence Synthesis
Source: Health Expect. 2025 Nov 26;28(6):e70495. doi: 10.1111/hex.70495 (PMC12657262; doi:10.1111/hex.70495)
Supplement: Supplementary file 6 — Appendix 6: GRADE‐CERQual assessment [ 27, 28, 29, 30, 31, 32]. [file HEX-28-e70495-s001.docx]

# **Appendix 6: GRADE-CERQual assessment [27-32]**

| **Finding** | **Contributing records** | **Methodological limitations** | **Coherence** | **Adequacy** | **Relevance** | **Overall** |
| --- | --- | --- | --- | --- | --- | --- |
| **Theme 1: Building a successful PPI partnership** | | | | | | |
| Researchers structured PPI activities to gain the involvement of parents as PPI contributors, such as meeting in the evenings, incorporating PPI activities into their routine schedules, meeting online or face-to-face in a familiar space, with varied success. | Levene *et al.* [37], Morgan *et al.* [38], Rayment *et al.* [41], Timm *et al*. [43]. | Serious concerns | No or very minor concerns | Moderate concerns | No or very minor concerns | Moderate confidence |
|  |  | **Reason(s):** Out of four records, two records did not provide adequate reporting of data analysis methods. One record did not provide adequate reporting of data collection methods.  Three out of four records did not report attempts to establish rigour; e.g. using triangulation, reflexivity, or respondent validation. | **Reason(s):** This finding was supported by the records. | **Reason(s):** Four records contributed to this finding, which had thin data. However, the finding is descriptive. | **Reason(s):**  One record was focused on ‘co-creation’ of an RCT. However, all contributing data was relevant to the QES aims. | **Reason(s):** Serious concerns regarding methodological limitations (-1) |
| Actions such as recruiting PPI contributors from pre-existing groups, such as a mother and baby groups, or using online PPI were found to be beneficial in reaching under-represented groups. | Levene *et al.* [37], Morgan *et al.* [38], Rayment *et al.* [41]. | Moderate concerns | Minor concerns | Moderate concerns | No or very minor concerns | Moderate confidence |
|  |  | **Reason(s):** Out of three records, one record did not provide adequate information on how data analysis was conducted. Two out of three records did not report attempts to establish rigour. | **Reason(s):** There was some data that differed, but this data was not explored by the record authors in detail (e.g. why their approach did not reach under-represented groups) | **Reason(s):** Three records contributed to this finding, which had thin data. However, the finding is descriptive. | **Reason(s):** All contributing records were reporting on PPI process and provided data on the approach taken by researchers to PPI recruitment, which is relevant to stakeholders’ experiences and perspectives of PPI. | **Reason(s):** Moderate concerns relating to both adequacy and methodological limitations, in addition to minor concerns regarding coherence (-1). |
| Researchers acknowledged that one PPI approach would not be suitable for all contexts and took a responsive and flexible approach to make involvement suit the needs of PPI contributors. | Morgan *et al.* [38], Onukwugha *et al.* [39], Rayment *et al.* [41], Timm *et al.* [43]. | Serious concerns | Minor concerns | Moderate concerns | Minor concerns | Low confidence |
|  |  | **Reason(s):** Two out of four records did not provide adequate information on data analysis methods. Two records did not report using not report attempts to establish rigour. | **Reason(s):** There was some variation in the data concerning the success of the approach taken to recruit and retain PPI contributors. | **Reason(s):** Four records contributed to this finding, which had thin data. However, the finding is descriptive. | **Reason(s):**  One record was focused on ‘co-creation’ of an RCT. The other included records aimed to provide an account of PPI conducted in the development of an intervention. | **Reason(s):** Serious concerns regarding methodological limitations (-1), in addition to moderate and minor concerns (-1) in the remaining 3 components. |
| Researchers encountered some difficulties ensuring that all PPI contributors were provided the opportunity to share their views while also achieving group consensus. Group tensions, dominant voices, and working with new PPI contributors who were unfamiliar with PPI were identified as challenges. | Levene *et al.* [37],Morgan *et al.* [38], Onukwugha *et al.* [39], Rayment *et al.* [41], Timm *et al.* [43] | Moderate concerns | No or very minor concerns | Moderate concerns | Minor concerns | Moderate confidence |
|  |  | **Reason(s):** Two records did not provide adequate information on data analysis methods. Three out of five records did not report attempts to establish rigour. | **Reason(s):** This finding is supported by the records. | **Reason(s):** Varied in levels of quality and information from each record that contributed to this finding. | **Reason(s):**  One record was focused on ‘co-creation’ of an RCT. Difficulties of PPI are told from perspective of researchers in all included records. | **Reason(s):** Two components assessed as having moderate concerns (-1). |
| Researchers considered that PPI contributors’ views were valued and held to be of equal value. | Moss *et al.* [36],  Morgan *et al.* [38], Onukwugha *et al.* [39],  Rayment *et al.* [40] | Moderate concerns | Moderate concerns | Moderate  concerns | No to very minor concerns | Low confidence |
|  |  | **Reason(s):** One out of four records did not provide enough information on data analysis methods. One record did not report attempts to establish rigour. | **Reason(s):** There was some variation in the data, particularly when comparing to the finding of researchers’ varied attitudes to the insights that PPI contributors have. | **Reason(s):** Four records contributed to this finding, which had thin data. However, the finding is descriptive. | **Reason(s):**  As researchers are stakeholders within PPI this finding is relevant to the aims of the QES. The aim of the included records were varied but are relevant to the QES question of experiences and perspectives of PPI. | **Reason(s):**  Downgraded as three components were assessed as having ‘moderate concerns’ (-2). |
| **Finding** | **Contributing records** | **Methodological limitations** | **Coherence** | **Adequacy** | **Relevance** | **Overall** |
| **Theme 2: Impact of PPI on trial design and development** | | | | | | |
| Researchers discussed the impact PPI had on improving the clarity and sensitivity of the language used within the trial, notably in participant information leaflets, to avoid causing anxiety or confusion for trial participants and parents. | Lammons *et al.* [35], Moss *et al.* [36], Levene *et al.* [37], Morgan *et al.* [38], Patel [40], Rayment *et al.* [41]. | Minor concerns | No to very minor concerns | No to very minor concerns | No to very minor concerns | High confidence |
|  |  | **Reason(s):** Appropriate data collection methods reported for all records. Four of six records did not adequately report attempts to establish rigour. One record did not meet any criteria for quality assessment (not applicable due to record type) | **Reason(s):** The finding was supported by the records. | **Reason(s):** Six records contributed to this study, of which five provided rich data compared to one record which contributed relatively thin data. | **Reason(s):**  One record was an opinion piece with an accompanying case study, but still provided data on perspectives of PPI in a neonatal trial. | **Reason(s):**  Minor concerns regarding methodological concerns, however the three remaining components have no to very minor concerns (-0). |
| Researchers reported that PPI resulted in reducing the burden and stress of trial participation; by ensuring adequate time to absorb trial information, and reducing the time or investment required to participate in the proposed trial. | Lammons *et al.* [35], Moss *et al.* [36], Levene *et al.* [37], Silver *et al.* [42], Timm *et al.* [43]. | Minor concerns | No to very minor concerns | No to very minor concerns | Minor concerns | Moderate confidence |
|  |  | **Reason(s):** Appropriate data collection for all included records. Varied issues with reporting of validity/ reliability across all records. | **Reason(s):** The finding was supported by the records. | **Reason(s):**  Five records contributed to this finding, and provided varied quality of information. | **Reason(s):**  One record was focused on ‘co-creation’ of an RCT. One record reported on involvement of a community advisory board which only included one father advocate (i.e. one PPI contributor). | **Reason(s):** Minor concerns regarding methodological concerns and minor concerns regarding relevance (-1). |
| PPI contributors incorporated a focus on the emotional and mental wellbeing of trial participants and families into the proposed trial design, to recognise the sensitivity of recruiting preterm babies or women who have potentially experienced traumatic pregnancies and births. | Lammons *et al.* [35], Moss et al. [36], Levene *et al.* [37], Morgan *et al.* [38], Rayment *et al.* [41], Silver *et al.* [42]. | Moderate concerns | No to very minor concerns | Minor concerns | Minor concerns | Moderate confidence |
|  |  | **Reason(s):** One record did not adequately report data analysis methods.  Varied issues with reporting of validity/ reliability across all records, with two not reporting any attempts to establish rigour. | **Reason(s):** The finding was supported by the records. | **Reason(s):** Records varied in terms of depth and quality of data. | **Reason(s):**  One record reported on involvement of a community advisory board which only included one father advocate (i.e. one PPI contributor) | **Reason(s):** Moderate concerns regarding methodological limitations and minor concerns regarding relevance (-1). |
| PPI reframed how the proposed trial was presented to potential participants; as a collaborative effort of both trial researchers and mothers or families, that viewed participants as individuals. | Lammons *et al.* [35], Moss *et al.*  [36], Levene *et al.* [37], Silver *et al.* [42]. | Moderate concerns | Minor concerns | Serious concerns | Minor concerns | Low confidence |
|  |  | **Reason(s):** Appropriate data collection methods reported for all records. Varied issues with reporting of validity/ reliability across all records, with one record not reporting any attempts to establish rigour. | **Reason(s):** The finding was supported by the records. However, there was some variation, with one record stating that PPI impact resulted in reduced decision-making for trial parents (viewed as benefit). | **Reason(s):** Data from records is thin. Two records reported data from the same PPI activity. | **Reason(s):**  One record reported on involvement of a community advisory board which only included one father advocate (i.e. one PPI contributor) | **Reason(s):** Downgraded as serious concern regarding data adequacy (-1) and moderate concerns with methodological limitations (-1) |
| Researchers considered that that PPI’s impact on the language used within the trial would improve trial recruitment. | Moss *et al.* [36], Patel [40], Rayment *et al.* [41]. | Serious concerns | No to very minor concerns | Moderate concerns | No to very minor concerns | Moderate confidence |
|  |  | **Reason(s):**  One record did not provide adequate reporting of data analysis methods. One record did not meet any criteria for quality assessment (not applicable due to record type). Issues relating to establishing reliability and validity for all three records. | **Reason(s):** The finding was supported by the records. | **Reason(s):**  Data is thin but this is a descriptive finding. | **Reason(s):** One of the records is a case study, however the protocol had allowed for grey literature to be included. | **Reason(s):** Downgraded by one level due to serious concerns of methodological limitations (-1). |
| Researchers perceived that PPI contributors provided them with unique and complex insights that could not be as easily accessed or replicated through qualitative research methods. | Levene *et al.* [37], Morgan *et al.* [38], Rayment *et al.* [41], Silver *et al.* [42]. | Moderate concerns | Minor concerns | Moderate concerns | Minor concerns | Moderate confidence |
|  |  | **Reason(s):**  One record did not adequately report data analysis. Two records lacked adequate reporting on establishing rigour. | **Reason(s):** Generally, researchers stated that contributors provided unique insights, but in some records, researchers had anticipated some of the feedback. | **Reason(s):** Data is thin but this is a descriptive finding. | **Reason(s):**  One record reported on involvement of a community advisory board which only included one father advocate (i.e. one PPI contributor). | **Reason(s):** Downgraded due to moderate concerns regarding methodological limitations and data adequacy (-1). |
| Researchers believed that PPI contributors reinforced and strengthened the relevancy of trial’s aims for the target population. | Lammons *et al.* [35], Levene *et al.* [37], Morgan *et al.* [38], Patel [40], Rayment *et al.* [41],  Silver *et al.* [42]. | Moderate concerns | No to very minor concerns | Moderate concerns | Minor concerns | Moderate confidence |
|  |  | **Reason(s):** One record did not meet any criteria for quality assessment (not applicable due to record type). Two additional records did not adequately report attempts to establish rigour.  One record did not adequately report data analysis. | **Reason(s):**  The finding was supported by the records. | **Reason(s):** Data is thin, but this is a descriptive finding. | **Reason(s):**  One record reported on involvement of a community advisory board which only included one father advocate (i.e. one PPI contributor) | **Reason(s):** Downgraded due to moderate concerns regarding methodological limitations and data adequacy (-1) |
| Researchers considered PPI contributors’ experiential knowledge was distinct from researchers’ professional knowledge of research, and influenced contributors’ understanding and interaction with the research process. | Morgan *et al.* [38], Rayment *et al.* [41] | Moderate concerns | Minor concerns | Moderate concerns | No to very minor concerns | Moderate confidence |
|  |  | **Reason(s):** Out of two records, one record did not adequately report data analysis methods. | **Reason(s):** There was some variation in the data concerning the attitudes of researchers towards perceived differences in thinking process , however this is reported in the QES. | **Reason(s):**  Data is thin and limited. | **Reason(s):** Both records were relevant to the QES. | **Reason(s):** Downgraded due to moderate concerns regarding methodological limitations and data adequacy (-1). |
| **Finding** | **Contributing study** | **Methodological limitations** | **Coherence** | **Adequacy** | **Relevance** | **Overall** |
| **Theme 3: Impact of PPI on stakeholders** | | | | | | |
| PPI was described as a mutually rewarding and productive experience for both researchers and PPI contributors. | Moss *et al.* [36], Onukwugha *et al.* [39], Patel [40]. | Moderate concerns | No to very minor concerns | Moderate minor concerns | No to very minor concerns | Moderate confidence |
|  |  | **Reason(s):**  One record did not meet any criteria for quality assessment (not applicable due to record type). One record did not adequately report on establishing rigour. | **Reason(s):** The finding was supported by the records. | **Reason(s):** Three records contributed to this finding and varied in quality and quantity of data. | **Reason(s):** One of the records is a case study, however the protocol had allowed for grey literature to be included. | **Reason(s):** Moderate concerns regarding methodological limitations and data adequacy (-1) |
| For some PPI contributors, the PPI experience was a source of pride and ignited their interest in  continued involvement in PPI or advocacy work. In other cases, PPI did not result in a continued involvement in PPI. | Morgan *et al.* [38], Onukwugha *et al.* [39], Rayment *et al.* [41], Timm *et al.* [43]. | Moderate concerns | No to very minor concerns | Moderate concerns | Minor concerns | Moderate confidence |
|  |  | **Reason(s):** Two records did not adequately describe data analysis methods. One record did not adequately describe data collection methods. Two records adequate reporting on establishing rigour. | **Reason(s):** The finding was supported by the records. | **Reason(s):** Three records contributed to this finding, which had thin data. However, the finding is descriptive. | **Reason(s):** One record reported on involvement of a community advisory board which only included one father advocate (i.e. one PPI contributor). | **Reason(s):** Downgraded due to moderate concerns regarding methodological limitations and data adequacy (-1) |
